# Radiological diagnosis of gallstone sigmoid ileus or coleus: Case report and literature review

**DOI:** 10.4102/sajr.v29i1.3149

**Published:** 2025-08-19

**Authors:** Suman Mewa Kinoo, Vanesha Naidu, Jaynund Maharajh

**Affiliations:** 1 Department of Surgery, Faculty of Medicine, University of KwaZulu-Natal, Durban, South Africa; 2 Department of Surgery, Victoria Mxenge Hospital, Durban, South Africa; 3 Department of Radiology, Faculty of Medicine, University of KwaZulu-Natal, Durban, South Africa; 4 Department of Radiology, Victoria Mxenge Hospital, Durban, South Africa

**Keywords:** gallstone ileus, gallstone sigmoid ileus, gallstone coleus, CT scan, plain radiograph

## Abstract

Gallstone ileus is a mechanical bowel obstruction caused by a gallstone within the intestinal tract. It occurs secondary to a fistulous communication between the gallbladder and intestine and commonly causes a small bowel obstruction termed gallstone ileus. It rarely causes a large bowel obstruction termed gallstone sigmoid ileus (GSI) or gallstone coleus.

**Contribution:**

A case of gallstone sigmoid ileus with typical plain radiography and CT scan findings and a review of different imaging modalities for this condition.

## Introduction

Gallstone ileus (GI) was first described by Thomas Bartholin in 1654.^1^ It is defined as the impaction of one or more gallstones (GS) anywhere within the gastrointestinal tract causing a mechanical intestinal obstruction.^2^ The term ‘ileus’ is therefore a misnomer as the obstruction is mechanical. It is an infrequent complication of cholelithiasis occurring in 0.3% – 0.5% of all patients with GS.^3^ The pathophysiological mechanism is a cholecysto-enteric fistulous communication between the gallbladder (GB) and the duodenum with impaction of the GS at the terminal ileum resulting in a small intestinal obstruction accounting for 60% – 85% of all GI cases.^4^ In approximately 2% of cases, the GS remains impacted at the site of the fistula in the duodenum and causes a gastric outlet obstruction (Bouveret’s syndrome).^4^ Rarely, the fistulous communication is between the gallbladder and the colon with impaction of the GS at the sigmoid colon causing a large bowel obstruction termed gallstone sigmoid ileus (GSI) or gallstone coleus and accounts for 4% of all GI cases.^4^ Plain abdominal radiography (AXR) consistent with Rigler’s triad, named after Leo George Rigler (American radiologist 1896–1979), is usually suggestive of the diagnosis and can be confirmed with CT scan.^5^ Management, most commonly, is operative, which involves enterotomy and removal of the obstructing stone, and addressing the cholecysto-enteric fistula in either a 1- or 2-stage operation.

### Ethical considerations

The patient provided written informed consent for the publication of this case report and the accompanying clinical images. All identifiable information, including the patient’s name and personal details, has been removed to protect privacy and maintain patient confidentiality.

## Patient presentation

**FIGURE 1:**
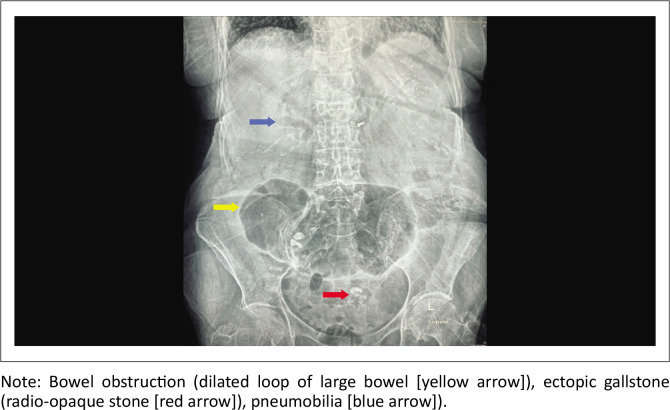
Plain supine abdominal radiograph demonstrating Rigler’s triad.

**FIGURE 2:**
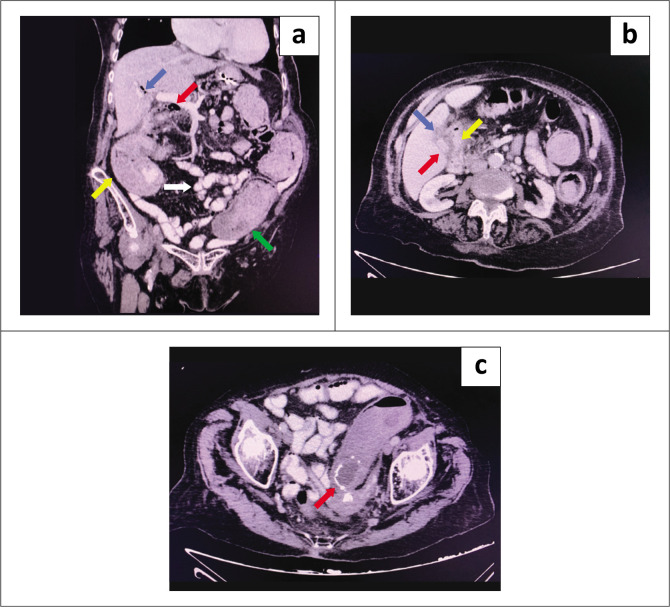
(a) Coronal contrasted CT scan demonstrating intrahepatic duct pneumobilia (blue arrow) and extrahepatic duct pneumobilia (red arrow), dilated ceacum (yellow arrow) and dilated large bowel proximal to sigmoid colon (green arrow) with non-dilated small bowel (white arrow) indicating a closed-loop bowel obstruction. (b) Axial contrasted CT scan demonstrating a shrunken gallbladder (red arrow) with surrounding fat stranding (blue arrow) in the region of the duodenum and hepatic flexure (yellow arrow). No obvious fistula was identified. (c) Axial contrasted CT scan demonstrating ectopic radio-opaque gallstone (oval low-density structure with rim calcification in the lumen of the sigmoid colon [red arrow]) with proximal dilatation of the sigmoid colon.

An 84-year-old woman presented with a 3-day history of constipation and abdominal distension. She was known to have atrial fibrillation (AF) on warfarin; however, she was not on any rate-controlling medication. Examination revealed a frail-looking lady, in fast AF with a pulse ranging between 130 and 150 beats per minute. She was otherwise haemodynamically stable with a blood pressure of 126/80 mmHg. She was mildly dehydrated but with normal urea and electrolytes. A thyroid function test (TFT), undertaken to exclude hyperthyroidism as a possible cause of her AF, was also normal. Her abdomen was distended but not peritonitic. The naso-gastric tube that was inserted had no drainage of contents. Abdominal radiography revealed a large centrally located dilated loop of large bowel with a suspicion of pneumobilia and a radio-opacity in the pelvis (Figure 1). Giving these findings, a GI/GSI was suspected but not confirmed, and a contrasted CT was performed. Computed tomography confirmed pneumobilia (Figure 2a) and features of fat stranding and inflammation involving the area of the duodenum and hepatic flexure of the colon, with a shrunken GB (Figure 2b). No obvious fistula was identified on CT. There was a closed-loop large bowel obstruction (indicated by a dilated caecum measuring 8.9 cm and a transition point distal to the radio-opaque ectopic stone in the sigmoid colon at the pelvic brim with no dilatation of the small bowel) (Figure 2a and Figure 2c). There was no evidence of pneumatosis coli on CT.

## Management and outcome

**FIGURE 3:**
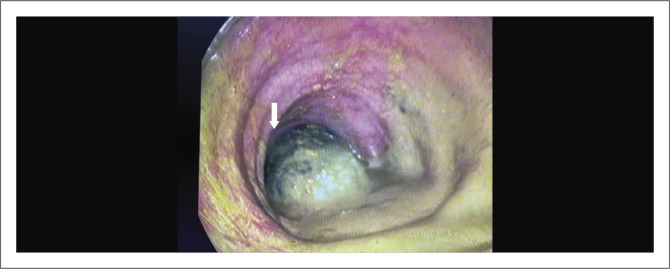
Sigmoidoscopy demonstrating the large ectopic gallstone in the sigmoid colon obstructing the lumen (white arrow).

**FIGURE 4:**
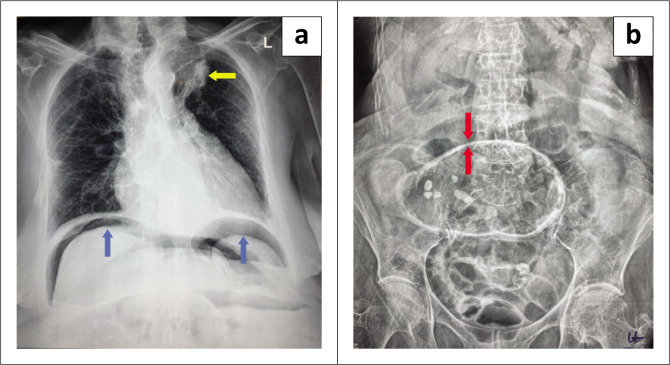
(a) Erect chest radiograph demonstrating free subcostal air (blue arrows) and a left paratracheal opacity causing right tracheal deviation (yellow arrow). (b) Supine abdominal radiograph demonstrating pneumoperitoneum, Rigler’s sign (red arrow).

**FIGURE 5:**
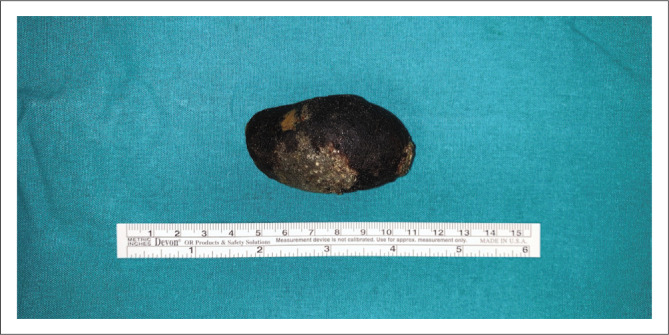
Gallstone measuring 68 mm in length and 40 mm in breadth.

Due to the patient’s age, uncontrolled rapid AF, and raised international normalised ratio (INR), warfarin was stopped, the patient was started on intravenous amiodarone, and non-operative stone extraction with a flexible sigmoidoscopy was attempted. At flexible sigmoidoscopy, lithotripsy and extraction of the GS were unsuccessful (Figure 3). Post flexible sigmoidoscopy, an erect chest radiograph (CXR) and supine AXR revealed pneumoperitoneum in the form of free air under the diaphragm (Figure 4a) and Rigler’s sign (Figure 4b). In addition, there was a left paratracheal opacity on CXR causing tracheal deviation to the right (Figure 4a). A retrosternal goitre was suspected but not clinically apparent and due to the emergent nature of the clinical situation, and a normal TFT on admission as part of the workup for her AF, no further imaging was undertaken, and the patient proceeded to emergency laparotomy. At laparotomy, the findings were that of a pinhole perforation in a compromised caecum due to the closed-loop obstruction and a large GS impacted in the sigmoid colon at the level of the pelvic brim. The closed-loop obstruction was secondary to a competent ileo-caecal valve. The GS (Figure 5) was ‘milked’ back into the caecum and a limited right hemicolectomy was performed with a primary anastomoses. Dissection of the fistula between the GB and intestine was not attempted as a two-stage procedure was planned given the clinical scenario; however, the duodenum and hepatic flexure of the colon were densely adherent to the shrunken GB. The patient was discharged well on day 7 post-surgery. On follow up, the patient was offered a CT neck and chest to further delineate the nature of the opacity causing tracheal deviation; the patient declined any further workup or treatment regarding her cholecysto-enteric fistula which was not addressed at the initial operation. The patient was subsequently lost to follow up.

## Discussion

There is a dearth of literature on GSI due to its rarity. In the largest systematic review of 38 cases of GSI by Farkas et al.,^6^ the highest incidence was noted among elderly women. Forty-seven per cent of patients and 59% of patients had co-morbid cardiovascular disease and diverticulosis, respectively.^6^ Most cases (87%) had cholecysto-colonic fistulae and the remainder (13%) cholecysto-duodenal fistulae. The majority of stones were impacted in the recto-sigmoid junction, mostly due to underlying chronic diverticulosis and diverticula strictures at the recto-sigmoid area. Due to most of these patients being elderly with cardiac comorbidities, non-operative stone retrieval was always opted as first-line management; however, was only successful in 10/38 cases (26%). CT was the most commonly used modality of investigation undertaken to confirm the diagnosis.^6^

Although seen in the reported case, Rigler’s triad consisting of bowel obstruction, ectopic GS and pneumobilia, is recognisable on AXR only in a small number of cases (14% – 53%).^7^ Only 10% – 20% of GS are visible on AXR as their radio-opacity depends on their degree of calcification.^8^ Pneumobilia is present only in half of the cases of GSI. Furthermore, due to its subtlety, pneumobilia is often missed and diagnosed only on retrospective analysis. Pneumobilia can also occur in sphincter of Oddi dysfunction, among other causes, and therefore its presence may not be confirmatory of a cholecysto-enteric fistula.^8^ A change in location of the GS on a subsequent AXR after physical examination is referred to as Rigler’s tetrad.^9^ The presence of 2 air fluid levels in the right upper quadrant (medial corresponding to the duodenal bulb and lateral to the gallbladder with air) is referred to as Rigler’s pentad.^9^ Although not routinely recommended due to the risk of perforation and peritonitis, the administration of rectal contrast can pass around the clear halo of the radiolucent GS mimicking the shape of a snake’s head (Forchet sign), or may pass from the colon to the gallbladder demonstrating the cholecysto-colic fistula (Petren sign).^7^

Contrast enhanced CT is the diagnostic modality of choice for the diagnosis of GI, having a specificity of 100%, a diagnostic accuracy of 99%, and a sensitivity of 90% – 93%.^10^ Its sensitivity for Rigler’s triad is 78%.^11^ Based on their study of CT in GI, Yu et al. defined CT diagnostic criteria as: ‘(1) small bowel obstruction; (2) ectopic gallstone; either rim-calcified or total-calcified; (3) abnormal GB with complete air collection, presence of air-fluid level, or fluid accumulation with irregular wall’.^11^ Computed tomography has the added advantage of making a rapid diagnosis with identification of complications of GSI/GI such as ischaemia and perforation, and thus aids in determining whether surgical or conservative management will be most appropriate, leading to a decrease in the high rate of morbidity and mortality rates associated with this condition. Computed tomography demonstrates the exact point of obstruction and cholecysto-enteric fistula. It also alerts the surgeon to the presence of further stones in the gastro-intestinal tract in 3% – 44% of patients that should be sought after and removed to prevent recurrence of obstruction.^3^ Recurrence can occur in 5% of cases, with 85% of recurrence cases documented within the first 6 months after the initial surgical intervention.^12^

Unlike GI where a cutoff stone size of 2.5 cm is likely to pass through the ileo-caecal valve, there is no cut off value for GSI to spontaneously pass.^6,13^ Nevertheless, it is recommended that all dimensions of the calculus be measured.^3^ GS density, size and section incrementation affect the detection rate on CT. There are varying degrees of detection as reported by Barakos et al., GS could appear densely (48.3%) or slightly (11.5%) calcified, as an area with a rim of increased density (21.8%), as an area of soft-tissue density (14.9%), or as an area of low density (3.4%).^14^ Twenty-five per cent of GS may be missed on CT due to their composition and isoattenuation relative to surrounding fluid.^14^ Gan et al. recommended subtle important clues in assessing true GS size which include: (1) faint radiolucency in or beyond the soft tissue area, (2) soft tissue density sign which is an area of soft tissue density rather than a fluid density surrounding the calcified rim of GS and (3) air crescent sign which is trapped compressed air around the GS in a dependent fashion that would otherwise rise to the anterior wall.^7,15^

Although abdominal ultrasonography (US) is the diagnostic modality of choice for GS with an efficacy of greater than 95%, its use in an acute abdomen with bowel obstruction is limited due to gas and fluid-filled dilated bowel.^16^ Specifically, to GI, despite there being evidence that experienced sonographers can demonstrate Rigler’s triad, and combining US with AXR can increase the sensitivity finding on AXR to 74%, CT sensitivity is still superior for making a diagnosis.^16,17^

The sensitivity for the diagnosis of GS within the biliary tree with magnetic resonance cholangio-pancreatography (MRCP) is 97.7%.^18^ MRI can also diagnose microlithiasis (< 3 mm) which can be missed on US.^18^ Although MRI sensitivity for Rigler’s triad is 100% which is higher than AXR (53%), AXR combined with US (74%), and CT (78%), MRI is time-consuming, and its use in the acute setting of intestinal obstruction is thus limited.^19^ Its use in GI/GSI is thus reserved for better delineation of the cholecysto-enteric fistula.

## Conclusion

Although rare, large bowel obstruction in an elderly woman should raise the suspicion of GSI. Computed tomography in suspected GSI is an invaluable tool in making a timely diagnosis and planning management and is thus the gold standard investigation.
